# Association between Breastfeeding Attitudes and Postpartum Depression among Mothers with Premature Infants during COVID-19 Pandemic

**DOI:** 10.3390/ijerph182010915

**Published:** 2021-10-17

**Authors:** Noor Fairuzi Suhana Yahya, Nur Islami Mohd Fahmi Teng, Najwa Shafiee, Norsham Juliana

**Affiliations:** 1 Faculty of Health Sciences, Universiti Teknologi MARA, Bandar Puncak Alam 42300, Selangor, Malaysia; fairuzisuhana@gmail.com (N.F.S.Y.); najwashafiee@gmail.com (N.S.); 2 Faculty of Medicine and Health Sciences, Universiti Sains Islam Malaysia, Nilai 71800, Negeri Sembilan, Malaysia; njuliana@usim.edu.my

**Keywords:** breastfeeding attitude, postpartum depression, mothers, premature infants, COVID-19

## Abstract

Breastfeeding is the best form of feeding for premature infants. However, mothers with premature delivery are frequently reported to be depressed, and this has been especially the case during the Coronavirus Disease-2019 (COVID-19) pandemic. We aimed to measure the level of breastfeeding attitude and its association with postpartum depression among mothers with premature infants in the Neonatal Intensive Care Unit (NICU) during the COVID-19 pandemic. A total of 248 mothers with a premature infant were observed in this cross-sectional study from the chosen NICUs of government hospitals in Selangor, Malaysia. The Iowa Infant Feeding Attitude Score (IIFAS) and the Edinburgh Postnatal Depression Scale, along with sociodemographic questionnaires, were used to obtain information on the mothers’ attitudes towards breastfeeding and the risk of postpartum depression. A higher percentage of mothers had a positive attitude towards breastfeeding (64.9%), with a mean IIFAS score of 66.30 ± 6.92. Meanwhile, about 27% of mothers with premature infants were reported to have high risk of depressive symptoms. Mothers with a high risk of depression were less likely to have a positive attitude towards breastfeeding (OR 0.37, 95% CI 0.199, 0.675) as compared to mothers with a low risk of depression (*p* < 0.01). We found that there is an association between the risk of depression and the attitude towards breastfeeding. Early identification of maternal mental health problems should be addressed to ensure the willingness of mothers to continue breastfeeding.

## 1. Introduction

Breast milk provides optimal nutrients to protect infants against various diseases such as upper and lower tract infections, as well as protecting against Type 1 diabetes and reducing asthma during infancy and beyond [1]. If the benefits for a full-term infant are enormous, a preterm infant may be entitled to far more benefits of breast milk. Unfortunately, premature infants are the population at risk where these infants are born before completing the maturation process that equips them for extrauterine life, particularly in the latter weeks of pregnancy [2]. Preterm birth rates are rising in most countries [3]; in Malaysia, the prevalence increased from 8% in 2010 to 12.4% in 2015 [4]. Hence, managing nutritional needs among premature infants by encouraging breastfeeding is mandatory to ensure reductions in physiological problems, despite the beneficial pharmacokinetics of several available drugs [2].

In addition, breastfeeding provides several advantages to mothers, including protection against breast and ovarian cancer, aid in postpartum recovery, and improved mother–infant bonding [1]. In Malaysia, the National Breastfeeding Policy has encouraged mothers to breastfeed their infants exclusively for the first six months and continue up to two years of age [5]. However, statistics in Malaysia showed a lower prevalence (only 47.1%) of mothers practising exclusive breastfeeding [6]. Previous studies have demonstrated that breastfeeding attitude is a significant determinant in explaining exclusive breastfeeding intention and behaviour [7,8,9]. Breastfeeding attitude, which encompasses belief and knowledge, is defined as women’s predisposition to express feelings, thoughts, and behaviours associated with a psychological mindset in developing their breastfeeding behaviour [10]. A previous study evidenced that mothers with a positive attitude are highly knowledgeable about breastfeeding [11] and intend to undertake actual and exclusive breastfeeding [12].

Nevertheless, it was reported that stressed and depressed mothers were lower in self attitude and self-efficacy towards breastfeeding [13,14]. This was especially concerning for mothers with preterm infants who needed to be hospitalised in the Neonatal Intensive Care Unit (NICU) and were commonly reported to have high perceived stress, anxiety, and depression [15]. As reported recently, factors associated with stress were the infant’s earlier gestational age [16], the infant’s appearance, the mother’s role and relationship with the infant, and the physical environment at the ward [17]. In addition, postpartum depression symptoms such as a continuous feeling of anxiety, unstable emotions, and inability to deal with a baby reduced the mothers’ breastfeeding attitude [18]. Hence, postpartum depression influences the mothers’ intention [19], self-efficacy [9,14], and behaviour with regard to breastfeeding [20]. As a result, it is believed that the mothers’ perceived stress or depression should be lowered to a level that will positively improve their breastfeeding attitude.

However, the current Severe Acute Respiratory Syndrome Coronavirus 2 (SARS-CoV-2) pandemic, also known as Coronavirus Disease-2019 (COVID-19) [21], was reported to influence the rate of breastfeeding and depression among postpartum mothers [22]. The fear of being contracting COVID-19 and transmitting it to the infants during hospitalisation, and the decrease in mother–infant contact due to policy restrictions [22,23,24], increased the anxiety and stress of mothers, which subsequently affected the breastfeeding rate. COVID-19 is a highly contagious infectious disease that can cause fever, cough, or respiratory illness, which may lead to mortality [25]. The first case of COVID-19 was discovered in Malaysia on 25th January 2020, and the rate of infected cases has continued to rise despite the government’s implementation of a non-pharmacological preventive approach [26]. However, the World Health Organisation (WHO) confirmed that breastfeeding during the COVID-19 pandemic could boost an infant’s immunity and outweigh the risk of infection, regardless of the mother’s state, including being infected or suspected to be infected with COVID-19 [27].

Therefore, it is critical to consider the factors associated with the attitude to breastfeeding and the assessment of depression status to assist primary healthcare providers in encouraging breastfeeding. Unfortunately, in our region, there is a lack of studies investigating factors associated with breastfeeding attitude and its relation to depression levels, primarily among mothers with premature infants, especially during the COVID-19 pandemic. Thus, this study aims to measure the level of breastfeeding attitude and its relationship with postpartum depression among mothers with premature infants in NICUs during the COVID-19 pandemic.

## 2. Materials and Methods

### 2.1. Research Design

This study is a cross-sectional study that was carried out in 2020 during the onset of COVID-19. The questionnaires were conducted to assess their breastfeeding attitude level and its associated factors, including depression status. The study was approved by the Medical Research & Ethics Committee, Ministry of Health, Malaysia, with the following approval number: NMRR-19-2583-50571(IIR).

### 2.2. Setting

The study was undertaken in Selangor, a state on the Western Coast of Peninsular Malaysia, due to its high number of preterm births (*n* = 1858) [4]. Therefore, the data were only collected in a few selected government hospitals with NICU wards, where most preterm infants were admitted.

### 2.3. Sample

The sample size was determined using the Krejcie and Morgan table [28], based on the nearest population size of preterm birth delivery from 2013 to 2015 in Selangor, Malaysia (*n* = 1858) [4], resulting in 237 samples. After considering the dropout rate of 5%, the required sample size, according to the calculation, was 249 samples [28]. A simple random sampling method was used, whereby each second mother who came to the NICU ward was selected as a participant. The inclusion criteria included mothers with an infant admitted to the NICU due to prematurity (below 37 weeks), postpartum mothers (from one week to six months after delivery), mothers aged between 19 and 40 years old, and Malaysian citizens. The exclusion criteria included mothers diagnosed with mental illnesses such as psychosis or bipolar disorder, or having a history of depression during or prior to the antenatal phase, as determined by a psychiatrist, to prevent confounding of the results. Illiterate mothers, either in Malay or English, were excluded from this study too. As Malaysia has three major ethnicities, Malay, Chinese and Indian, mothers who are non-Malay might not be adequately literate in Malay. Hence, for non-Malay speakers, English was used during the data collection process.

### 2.4. Measurement

In this study, self-administered questionnaires were completed to address three subsets of questions, which were (1) participant characteristics involving the mother’s age, ethnicity, education, occupation, and household income per month, and the infant’s gestational age; (2) the Iowa Infant Feeding Attitude Scale (IIFAS); and (3) the Edinburgh Postpartum Depression Scale (EPDS). Each questionnaire used in this study was described in detail, as shown below:

#### 2.4.1. Infant Feeding Attitude Scale (IIFAS)

The Infant Feeding Attitude Scale (IIFAS) was developed by De La Mora and Russell [29], and was designed to measure the attitude of mothers towards infant feeding. The scale has 17 items assessed on a 5-point Likert scale (strongly disagree, disagree, neutral, agree, and strongly agree). The items inquire about the mother’s view and perceptions on infant feeding. Cost, nutrition, and infant bonding are some of the variables that are measured. Items that are asterisked are reverse scored, and indicate the items in favour of formula feeding (1 = 5, 2 = 4, 4 = 2, 5 = 1). The minimal score of the overall scale is 17, which demonstrates favourable attitudes towards formula feeding, and the highest score of 85 demonstrates favourable attitudes towards breastfeeding. A higher level of breastfeeding attitude is determined based on the higher score of 65 and above, while a lower level of breastfeeding attitude is indicated by a lower score than 65 [30]. The IIFAS scale has been used extensively, with Cronbach’s alpha ranging from 0.72 to 0.87, demonstrating it to be a valid and reliable instrument [12,31,32]. For the Malaysian population, the Malay version of IIFAS has been translated and validated by local researchers [7,8]. Thus, reliability was assured in using the Malay version of the IIFAS in this study.

#### 2.4.2. Edinburgh Postpartum Depression Scale

The Edinburgh Postnatal Depression Scale (EPDS) is a self-rating scale designed to assess depressive symptoms, especially among postpartum mothers [33]. The tool has ten items and a 4-point Likert-type scale with values between 0 and 3 that indicate the symptom’s severity for the last seven days. The total scores are calculated by adding the individual scores for the ten items, with higher scores representing a greater number and frequency of depressive symptoms. The validity and reliability of the Malay version of EPDS were confirmed by Mahmud et al. [34], with the internal consistency designated at 0.86. The EPDS cut-off point was calculated as 12, where mothers who had scored more than 12 were considered to have a higher degree of depressive symptoms [34,35]. The author obtained the original translated questionnaire from Arifin et al. [35] and used the tools with Arifin’s permission.

### 2.5. Data Collection

Participants were enrolled between February and August 2020. Data were collected via self-administered questionnaires, and the researcher aided those who were unable to complete the questionnaires due to being preoccupied with their infants. The objectives of the study and the confidentiality of the data were expressed to all the respondents, and they were also asked to sign consent forms upon giving agreement to participate. Participants were also informed that they could withdraw at any moment during the study. Therefore, the participants completed the questionnaires only after providing their consent. The data collection was conducted in the NICUs, targeting mothers who were, at that time, visiting their infants, or mothers who were assisting their infants in the inpatient ward.

### 2.6. Data Analysis

Data were analysed using the IBM Statistical Package for Social Science (SPSS) version 23 (SPSS Inc., Chicago, IL, USA). Descriptive analyses were conducted to identify the frequencies for the categorical variables, and the mean and standard deviation for the continuous variables. One-way analysis of variance and the independent-sample t-test were used to assess the differences in IIFAS scores between particular groups. Simple logistic regression was used to determine the significance of the variables, with a *p*-value < 0.25 being the threshold for inclusion in the multiple logistic regression [7]. Further analysis, using multiple logistic regression, was conducted to calculate the odds ratio and associated 95% confidence interval for predictors of breastfeeding attitude among mothers with premature infants in NICUs in Selangor. A *p*-value of 0.05 was considered statistically significant.

## 3. Results

### 3.1. Characteristics of the Participants

A total of 248 participants were recruited in this study upon giving their consent. Table 1 shows the characteristics of the mothers and the infants. The majority of the mothers were between the ages of 19 and 34 (70.2%), with a mean age of 31.41 ± 5.86 years, were Malay (87.5%), were educated up to the level of secondary education or below (44.4%), and were working in the non-government sector (56.4%). The infant characteristics showed that a majority of mothers had their infant in the late preterm period (52.8%) and underwent vaginal delivery during the process of giving birth (56.9%), and a majority of infants weighed more than 2500 g at birth (65.3%).

### 3.2. Breastfeeding Attitude Status

Overall, most mothers (64.9%) had a high breastfeeding attitude score (score ≥ 65), with the mean IIFAS score being 66.30 ± 6.92. Based on the IIFAS questionnaire, the majority of participants agreed that breastfeeding could increase mother–infant bonding (96.4%) and is more convenient than formula feeding (91.5%), and that breast milk is an ideal food for babies (93.5%) and is more easily digested than formula milk (91.5%) (Table 2). In terms of economy, most participants (85.9%) agreed that breast milk is less expensive than formula milk. However, only half of the participants believed that the nutritional benefits of breast milk will last longer (even after the baby is weaned from breast milk) (57.3%), that breastfeeding is a better choice even when the mother has started work (60.9%), and that women can breastfeed in public places (51.6%). In contrast, very few participants agreed that the mother can breastfeed her baby if she occasionally drinks alcohol (15.7%).

### 3.3. Characteristics of Participants and Their Association with Breastfeeding Attitude

Table 3 shows the mothers’ and infants’ characteristics and their association with breastfeeding attitude. Based on univariate analysis, the results demonstrated that among all the characteristics, only maternal occupational status and depression status had a significant association (*p* < 0.005) with breastfeeding attitude. Non-government workers (OR 0.42, 95% CI 0.18–0.99) and non-working mothers (OR 0.38, 95% CI 0.15–0.94) were less likely to have a positive attitude towards the practice of breastfeeding. Nonetheless, mothers with a higher risk of depressive symptoms were less likely to have a positive breastfeeding attitude (OR 0.438, 95% CI 0.25–0.78) than mothers with a lower risk of depressive symptoms. However, other sociodemographic factors such as age, ethnicity, marital status, education, and BMI, and infant-related factors such as the method of delivery, birth weight, and gestational age, did not show any significant association with breastfeeding attitude.

### 3.4. Breastfeeding Attitude, Depression Status, and Its Associated Factors

Depression status indicated that 27% of the mothers had a higher risk of depression, with the mean EPDS score being 8.14 ± 6.00. When comparing breastfeeding attitudes according to depression status, mothers with a higher risk of depression had significantly (*p* < 0.001) lower breastfeeding attitude scores (63.73 ± 6.69) as compared to mothers with a lower risk of depression (67.25 ± 6.78) (Table 4). Further analysis of logistic regression was conducted to confirm the apparent association between depression and occupational status and breastfeeding attitude, as shown in Table 5. According to stepwise logistic regression analysis, the results consistently showed that mothers with a higher risk of depression were less likely to have a positive attitude towards breastfeeding (OR 0.37, 95% CI 0.19–0.68), followed by non-government workers and (OR 0.38, 95% CI 0.16–0.91), and non-working mothers (OR 0.27; 95% CI 0.11–0.72).

## 4. Discussion

Although research documenting breastfeeding attitudes has previously been published in various settings in Malaysia [10,11,12], to the best of our knowledge, this is the first study in Malaysia to employ a validated IIFAS scale to measure the breastfeeding attitudes of mothers with premature infants in NICUs during the COVID-19 pandemic. It was found that the average score for breastfeeding attitude (66.30 ± 6.92) is above the cut-off point (≥65), indicating that these mothers had a positive attitude towards breastfeeding. A local study [36] conducted among Malay women with healthy infants in Ampang district found similar results (66.10 ± 8.11), indicating that these mothers had neutral-to-positive attitudes towards breastfeeding. On average, mothers’ attitudes towards breastfeeding in Malaysia were quite satisfactory. However, the United Kingdom (70.6 ± 6.47) [37] and Spain (69.76 ± 7.75) [12] reported higher scores than the present study. A higher level of education among the mothers in those studies [12,37] compared to the present study might explain the variation. Here, a higher level of education demonstrates a better breastfeeding attitude. Furthermore, various cultural elements contribute to the differences in breastfeeding attitude. For example, restraining alcohol consumption during breastfeeding is prevalent among Muslim mothers. Thus, only a few mothers agreed that “A mother who occasionally drinks alcohol should breastfeed her baby” (as shown in Table 2). In addition, Ishak et al. [38] reported that the intention of their feeding method was to distinguish between the mothers’ attitudes. Mothers with higher breastfeeding intention had a higher breastfeeding attitude score (64.07 ± 6.16) compared to mothers who preferred formula feeding (59.50 ± 7.55) [38]. Thus, this indicates that a positive breastfeeding attitude is essential for assisting the mothers in terms of breastfeeding intention and subsequently enhancing their breastfeeding practices for a longer duration.

One of the factors that negatively affect breastfeeding attitude is maternal stress or depression [13]. This study confirms that mothers with a higher risk of depressive symptoms were more likely to have a negative attitude towards breastfeeding. This is in good agreement with other studies. For instance, Duran et al. [13] found that higher stress perceived by the mothers decreased the breastfeeding attitude among Turkish mothers. A study of 85 mother–infant dyads in India also showed a similar finding, where higher depression and anxiety scores among mothers with infants who had low birth weights were associated with poorer breastfeeding attitudes [39]. Furthermore, Khodabandeh et al. [18] sampled 200 mothers from rural and urban health centres in Iran with postpartum depression. They found that the mothers with higher levels of depression and anxiety had a more negative attitude towards breastfeeding. This finding is also in line with a study conducted among Nigerian mothers (*n* = 187), which indicates that depressive symptoms during the postpartum period and higher employment status were associated with negative attitudes towards breastfeeding [40]. Thus, these results strengthened the fact that poor psychosocial health is also one of the essential factors that can induce negative breastfeeding attitudes, which indirectly contribute to earlier breastfeeding cessation among postpartum mothers [9].

Various feeding methods, either by means of naso- or orogastric tube delivery prior to progressing to the oral feeding of premature infants in NICUs, may increase the risk of breastfeeding difficulties and stress among mothers [15,41]. It was suggested that the physiological response that occurs due to depression reduces oxytocin levels, which can disrupt the reflex of milk discharge from the breast and, in turn, induce early breastfeeding cessation [42]. Therefore, the incremental level of stress perceived by the mothers decreased the production of breast milk, which indirectly reduced their confidence and attitude towards breastfeeding. Nevertheless, the relationship between breastfeeding and depression is bidirectional, with either postpartum depression decreasing the rate of breastfeeding, or disengaging in breastfeeding increasing the risk of depression [43]. Not engaging in breastfeeding reduces the attenuating cortisol stress response, a factor that is related to higher depressive symptoms [44]. This relationship is evidenced by a recent study that showed that mothers with early negative breastfeeding experiences were 7.58-fold more likely to suffer from postpartum depression [45]. Furthermore, Khalifa et al. [46] demonstrated that an exclusive breastfeeding practice reduced the odds of postpartum depression by 80% among a sample of 300 Sudanese women. Thus, findings in the literature have shown that poor breastfeeding behaviour can also be associated with higher depression symptoms.

Globally, the recent COVID-19 pandemic has increased the prevalence of postpartum mothers having depression, as reported in China (30%) [47], Turkey (34%) [22], Mexico (29.2%) [48], Canada (40.7%) [49], and the United Kingdom (32.8% to 47.5%) [50]. Mothers with premature infants in NICUs also reported a higher prevalence of postpartum depression (12–68%), even before the pandemic [51]. In this present study, the prevalence of postpartum depression among mothers with premature infants is relatively high, with 27% of the participants experiencing depressive symptoms in this COVID-19 era. In comparison to the current study, Ong et al. [15] found that Malaysian mothers with premature infants had a higher prevalence of stress (56.5%) before the COVID-19 pandemic. However, Ong et al. [15] employed different tools in assessing stress conditions compared to the present study. The traumatic events from the preterm birth, the stressful and frightening emotions that arise due to the infant’s health condition, distress about being infected with COVID-19, and fear of COVID-19 transmission to the baby during breastfeeding are among the factors that contribute to the mother’s depression [14,22,52]. In fact, the strict standard operating procedures, such as travel restrictions and modification of hospital policies to reduce COVID-19 transmission, also increased the perceived stress of mothers [23]. Furthermore, some mothers intended to discontinue breastfeeding due to concerns about breastfeeding safety and a lack of face-to-face professional support [53]. All of these issues might influence a mother’s attitude towards breastfeeding. Hence, it is essential for NICU staff to provide adequate advice and support in terms of breastfeeding during hospitalisation in order to enhance the mother’s breastfeeding attitude and practice.

Recent literature has also agreed that breastfeeding attitudes are associated with sociodemographic factors. Surprisingly, non-working mothers in the present study were highly associated with reduced likelihoods of having positive breastfeeding attitudes. This finding is similar to that of Pilus et al. [7], who mentioned that unemployed mothers were less likely to exclusively breastfeed than their counterparts. The lower attitude to breastfeeding scores may be due to the lack of knowledge of the nutritional benefits of breast milk, the lack of opportunities to seek information due to having a small social network, and social constraints and traditions among the non-working mothers [7]. This result contrasts with another study, which reported that housewives were two times more likely to have positive breastfeeding intentions than working mothers [54]. Furthermore, the present result showed that mothers working in non-government sectors were less likely to have a positive breastfeeding attitude. Inadequate breastfeeding facilities at the workplace, length of maternity leave, and the absence of maternity policies in organisations were among the factors that influenced the discontinuation of breastfeeding [55,56]. Other factors such as ethnicity, age, household income, maternal education level, and infant birth weight were not associated with breastfeeding attitudes in this study, contradicting previous literature [13,18,36].

This study achieved its strength when the result showed that an optimum level of psychosocial well-being contributes to a better attitude towards breastfeeding. Social support by nearest kin such as husbands, mothers, or siblings is necessary to assist the mothers’ self-efficacy and confidence in continuing breastfeeding. Furthermore, this study can be used as evidence for health professionals or authorities in developing awareness campaigns on the benefits of breast milk. This ensures that mothers with a premature infant produce enough milk to initiate early breastfeeding during the crucial first few weeks of premature infants and continue breastfeeding even after discharge [57].

However, several limitations were also considered in this study. First, as this study only recruited mothers with premature infants, generalisation of the results to all postpartum mothers in Malaysia may not be appropriate. Secondly, because this study was conducted during the COVID-19 pandemic, several restrictions were imposed during data collection, such social distancing with the participants or limited time for collecting data in the NICUs, resulting in a smaller sample size being obtained for this study. Third, our study did not consider the intention or duration of breastfeeding. Thus, we cannot determine the causal effects of the breastfeeding attitude. Because of these limitations, additional studies with a larger sample size, other sociodemographic characteristics and breastfeeding determinants are needed to explore more of the attitudes towards breastfeeding held by Malaysian mothers, and their levels of psychosocial health.

## 5. Conclusions

Most of the mothers with premature infants in this study favoured the breastfeeding method compared to the formula feeding method. However, this study shows that having a high risk of depressive symptoms, being a non-government worker, and being a non-working mother were all predictors of having a negative attitude towards breastfeeding. Therefore, educational programs for postpartum mothers should be implemented to equip these mothers with good breastfeeding attitudes. Furthermore, mental health screening on postpartum mothers, particularly among mothers with premature infants, will help to identify highly depressed mothers, who can then be referred to competent healthcare professionals to improve their quality of life.

## Figures and Tables

**Table 1:** Participant’s characteristics (*n* = 248).

Variable	Mean (SD)	Frequency, *n* (%)
**Age**	31.41 ± 5.86	
19–34 years old		174 (70.2)
≥35 years old		74 (29.8)
**Ethnicity**		
Malay		217 (87.5)
Non-Malay		31 (12.5)
**Educational level**		
Secondary or lower		110 (44.4)
Diploma or apprentice		65 (26.2)
Higher education		73 (29.4)
**Occupation**		
Government		40 (16.1)
Private		140 (56.5)
Housewife/Not		
working		68 (27.4)
**Monthly household income**	RM ^a^ 5162.12 ± RM ^a^ 3917.87	
≤RM ^a^ 3000		91 (36.7)
RM ^a^ 3001–RM ^a^ 5000		64 (25.8)
≥RM ^a^ 5001		93 (37.5)
**Gestational age**	33.06 ± 3.30 weeks	
<34 weeks		
(extreme to moderate		
preterm)		117 (47.2)
34 weeks and above		
(Late preterm)		131 (52.8)
**Type of delivery**		
Normal delivery		141 (56.9)
Cesarean section		107 (43.1)
**Infant’s birth weight**		
<2500 g		86 (34.7)
≥2500 g		162 (65.3)
**Infant’s birth order**		
First baby		108 (43.5)
Others		140 (56.5)

^a^ RM = Ringgit Malaysia, is the currency of Malaysia.

**Table 2:** Attitudes towards breastfeeding among mothers of premature infants in Neonatal Intensive Care Units (*n* = 248).

Attitudes towards Breastfeeding	IIFAS Score	Agree ^b^		Neutral		Disagree ^c^	
	Mean ± SD	*n*	(%)	*n*	(%)	*n*	(%)
^a^ The nutritional benefits of breast milk last only until the baby is weaned from breast milk.	3.47 ± 1.58	142	57.3	23	9.3	83	33.5
2. ^a^ Formula-feeding is more convenient than breastfeeding.	4.59 ± 0.91	224	90.3	13	5.2	11	4.4
3. Breastfeeding increases mother–infant bonding.	4.83 ± 0.66	239	96.4	3	1.2	6	2.4
4. ^a^ Breast milk is lacking in iron.	4.45 ± 0.96	207	83.5	28	11.3	13	5.2
5. Formula-fed babies are more likely to be overfed than breastfed babies.	2.86 ± 1.44	79	31.9	65	26.2	104	41.9
6. ^a^ Formula-feeding is the better choice if a mother plans to work outside the home.	3.65 ± 1.39	151	60.9	46	18.5	51	20.6
7. Mothers who formula-feed miss one of the great joys of motherhood.	3.26 ± 1.58	120	48.4	38	15.3	90	36.3
8. ^a^ Women should not breastfeed in public places such as restaurants.	4.06 ± 1.52	128	51.6	34	13.7	86	34.7
9. Babies fed breast milk are healthier than babies who are fed formula.	4.06 ± 1.51	175	70.6	30	12.1	43	17.3
10. ^a^ Breastfed babies are more likely to be overfed than formula-fed babies	3.16 ± 1.45	110	44.4	62	25.0	76	30.6
11. ^a^ The father feels left out if a mother breastfeeds.	4.23 ± 1.15	198	79.8	26	10.5	24	9.7
12. Breast milk is the ideal food for babies.	4.68 ± 0.80	232	93.5	7	2.8	9	3.6
13. Breast milk is more easily digested than formula.	4.64 ± 0.91	227	91.5	9	3.6	12	4.8
14. ^a^ Formula is as healthy for an infant as breastmilk.	4.04 ± 1.15	182	73.4	44	17.7	22	8.9
15. Breast-feeding is more convenient than formula feeding.	4.59 ± 0.90	227	91.5	9	3.6	12	4.8
16. Breast milk is less expensive than formula.	4.44 ± 1.20	213	85.9	8	3.2	27	10.9
17. ^a^ A mother who occasionally drinks alcohol should not breastfeed her baby.	2.08 ± 1.38	39	15.7	43	17.3	166	66.9

^a^ This was reversed when calculating the score (Item: 1, 2, 4, 6, 8, 10, 11, 14, 17). ^b^ The ‘agree’ classification includes ‘strongly agree’ and ‘agree’. ^c^ The ‘disagree’ classification includes ‘strongly disagree’ and ‘disagree’.

**Table 3:** Association between participant’s background and positive breastfeeding attitude (*n* = 248).

Factors	Low Level ^a^ *n* (%) (*n* = 87)	High Level ^b^ *n* (%) (*n* = 161)	Crude OR (95% CI) ^c^	*p*-Value
**Maternal age (years)**				
18–34 years old	64 (73.6%)	110 (68.3%)	1.00	0.390
≥35 years old	23 (26.4%)	51 (31.7%)	0.775 (0.434, 1.385)	
**Maternal ethnicity**				
Malay	74 (85.1%)	143 (88.8%)	1.000	0.394
Non-Malay	13 (14.9%)	18 (11.2%)	1.396 (0.648, 3.004)	
**Marital status**				
Married	83 (95.4%)	160 (99.4%)	1.000	0.070
Others	4 (4.6%)	1 (0.6%)	0.13 (0.014, 1.179)	
**Maternal education level**				
Secondary or lower	41 (47.1%)	69 (42.9%)	0.84 (0.49, 1.42)	0.519
Diploma or higher education	46 (52.9%)	92 (57.1%)	1.00	
**Maternal occupation status**				
Government	8 (9.2%)	32 (19.9%)	1.000	
Non-government	52 (59.8%)	88 (54.7%)	0.42 (0.18, 0.99)	0.047 *
Not working	27 (31.0%)	41 (25.5%)	0.38 (0.15, 0.94)	0.038 *
**Maternal household income**				
≤RM3000	38 (43.7%)	53 (32.9%)	1.00	
RM 3001–RM 5000	21 (24.1%)	43 (26.7%)	1.47 (0.75, 2.86)	0.260
**Types of delivery**				
Normal delivery	53 (60.9%)	88 (54.7%)	1.00	
Cesarean section	34 (39.1%)	73 (45.3%)	1.293 (0.76, 2.19)	0.343
**Birth weight**				
<2500 g	30 (34.5%)	56 (34.8%)	1.013 (0.59, 1.75)	0.962
≥2500 g	57 (65.5%)	105 (65.2%)	1.00	
**Infant’s birth order**				
First baby	42 (48.3%)	66 (41.0%)	1.00	
Others	45 (51.7%)	95 (59.0%)	1.343 (0.79, 2.27)	0.270
**Gestational age**				
<34 weeks	42 (48.3%)	75 (46.6%)	1.00	
≥34 weeks	45 (51.7%)	86 (53.4%)	1.07 (0.64, 1.80)	0.799
**Depression status**				
Low risk	54 (62.1%)	127 (78.9%)	1.00	
High risk	33 (37.9%)	34 (21.1%)	0.438 (0.25, 0.78)	0.005 *

* Significant value at *p* < 0.05. ^a^ Low level = score < 65. ^b^ High level = score ≥ 65. ^c^ Simple Logistic Regression.

**Table 4:** Mean score of breastfeeding attitude and its associated factors among mothers with premature infants in Neonatal Intensive Care Units (NICUs) (*n* = 248).

Variable	Breastfeeding Attitude	
	Mean ± SD	*p*-Value
**Depression status**		*p* < 0.001 **
Higher risk (*n* = 67)	63.73 ± 6.69	
Lower risk (*n* = 181)	67.25 ± 6.78	
**Occupational status**		0.163
Government (*n* = 40)	68.10 ± 6.04	
Non-government (*n* = 140)	66.17 ± 7.08	
Not working (*n* = 68)	65.51 ± 6.98	

** Significant value at *p* < 0.001.

**Table 5:** Factors associated with positive breastfeeding attitude from stepwise logistic regression analysis (*n* = 248).

Variable	*n* (%)	Adjusted OR (95% CI) ^a^	*p*-Value
**Depression status**			
Lower risk	181 (73.0%)	1.00	
Higher risk	67 (27.0%)	0.37 (0.19, 0.68)	*p* < 0.001 **
**Maternal occupation status**			
Government	40 (16.1%)	1.00	
Non-government	140 (56.5%)	0.38 (0.16,0.91)	0.029 *
Not working	68 (27.4%)	0.27 (0.11, 0.72)	0.008 *

* Significant value at *p* < 0.05. ** Significant value at *p* < 0.001. ^a^ Multiple Logistic Regression.

## Data Availability

The data presented in this study are available on request from the corresponding author.

## References

[1] Subramanian V. Relevance of National Breastfeeding Policy (NBP) in Malaysian Society. Proceedings of the International Social Development Conference 2014.

[2] Machado J.S., Ferreira T.S., Lima R.C.G., Vieira V.C., de Medeiros D.S. (2021). Premature Birth: Topics in Physiology and Pharmacological Characteristics. Rev. Assoc. Med. Bras..

[3] Walani S.R. (2020). Global Burden of Preterm Birth. Int. J. Gynecol. Obstet..

[4] Jeganathan R., Karalasingam S.D., National Obstetrics Registry (NOR) (2017). 4th Report of National Obstetric Registry (Jan 2013–Dec 2015).

[5] National Coordinating Committee on Food and Nutrition (NCCFN) (2016). National Plan of Action for Nutrition of Malaysia III 2016–2025.

[6] Institute of Public Health (IPH) (2016). National Health and Morbidity Survey 2016: Maternal and Child Health (MCH).

[7] Pilus F.M., Ahmad N., Mohd Zulkefli N.A. (2019). Predictors of Exclusive Breastfeeding among Mothers Attending Rural Health Clinics in Hulu Langat District. Malays. J. Med. Health Sci..

[8] Bariah S., Hamid A., Yahya N. (2018). Determinants of Breastfeeding Behaviour. J. Clin. Health Sci..

[9] Mercan Y., Selcuk K.T. (2021). Association between Postpartum Depression Level, Social Support Level and Breastfeeding Attitude and Breastfeeding Self-Efficacy in Early Postpartum Women. PLoS ONE.

[10] Casal C.S., Lei A., Young S.L., Tuthill E.L. (2017). A Critical Review of Instruments Measuring Breastfeeding Attitudes, Knowledge, and Social Support. J. Hum. Lact..

[11] Khasawneh W., Kheirallah K., Mazin M., Abdulnabi S. (2020). Knowledge, Attitude, Motivation and Planning of Breastfeeding: A Cross-Sectional Study among Jordanian Women. Int. Breastfeed. J..

[12] Cotelo M.D.C.S., Movilla-Fernández M.J., Pita-García P., Novío S. (2018). Infant Feeding Attitudes and Practices of Spanish Low-Risk Expectant Women Using the IIFAS (Iowa Infant Feeding Attitude Scale). Nutrients.

[13] Duran S., Kaynak S., Karadaş A. (2019). The Relationship between Breastfeeding Attitudes and Perceived Stress Levels of Turkish Mothers. Scand. J. Caring Sci..

[14] Didarloo A., Rahmatnezhad L., Sheikhi S., Khodai F. (2017). Relationship of Spiritual Health and Perceived Stress with Breastfeeding Self-Efficacy: A Survey on Mothers with Hospitalised Neonates. Int. J. Pediatr..

[15] Ong S.L., Abdullah K.L., Danaee M., Soh K.L.G.L., Soh K.L.G.L., Japar S. (2019). Stress and Anxiety among Mothers of Premature Infants in a Malaysian Neonatal Intensive Care Unit. J. Reprod. Infant Psychol..

[16] Çekin B., Turan T. (2018). The Stress Levels of Parents of Premature Infants and Related Factors in Nenoatal Intensive Care Units. Turk. J. Pediatr..

[17] Lim C.J., Jayah K.P., Soon L.K. (2017). Parental Stress and Its Influencing Factors in the Neonatal Intensive Care Unit. Int. J. Public Health Clin. Sci..

[18] Khodabandeh M., Kashani L., Pirastehfar Z., Dargahloo S.D., Tabarestani M., Dordeh M., Rohni A., Heydari O., Ghazanfarpour M. (2020). Relationship between Attitude towards Breastfeeding and Postpartum Depression in Kerman, Iran. Int. J. Pediatr..

[19] Gila-Díaz A., Carrillo G.H., de Pablo Á.L.L., Arribas S.M., Ramiro-Cortijo D. (2020). Association between Maternal Postpartum Depression, Stress, Optimism, and Breastfeeding Pattern in the First Six Months. Int. J. Environ. Res. Public Health.

[20] Mikšić Š., Uglešić B., Jakab J., Holik D., Milostić Srb A., Degmečić D. (2020). Positive Effect of Breastfeeding on Child Development, Anxiety, and Postpartum Depression. Int. J. Environ. Res. Public Health.

[21] World Health Organization (WHO) Timeline of WHO’s Response to COVID-19. https://www.who.int/emergencies/diseases/novel-coronavirus-2019/interactive-timeline#!.

[22] Guvenc G., Yesilcinar İ., Ozkececi F., Öksüz E., Ozkececi C.F., Konukbay D., Kok G., Karasahin K.E. (2021). Anxiety, Depression, and Knowledge Level in Postpartum Women during the COVID-19 Pandemic. Perspect. Psychiatr. Care.

[23] Doyle F.L., Klein L. (2020). Postnatal Depression Risk Factors: An Overview of Reviews to Inform COVID-19 Research, Clinical, and Policy Priorities. Front. Glob. Women’s Health.

[24] Lemmon M.E., Chapman I., Malcolm W., Kelley K., Shaw R.J., Milazzo A., Cotten C.M., Hintz S.R. (2020). Beyond the First Wave: Consequences of COVID-19 on High-Risk Infants and Families. Am. J. Perinatol..

[25] Velavan P.C.G.M. (2020). The COVID-19 Epidemic. Trop. Med. Int. Health.

[26] Salman A.M., Ahmed I., Mohd M.H., Jamiluddin M.S., Dheyab M.A. (2021). Scenario Analysis of COVID-19 Transmission Dynamics in Malaysia with the Possibility of Reinfection and Limited Medical Resources Scenarios. Comput. Biol. Med..

[27] World Health Organization (WHO) Breastfeeding and COVID-19. https://www.who.int/publications/i/item/WHO-2019-nCoV-Sci_Brief-Breastfeeding-2020.1.

[28] Krejcie R.V., Morgan D.W. (1970). Determining Sample Size for Research Activities. Educ. Psychol. Meas..

[29] De La Mora A., Russell D.W., Dungy C.I., Losch M., Dusdieker L. (1999). The Iowa Infant Feeding Attitude Scale: Analysis of Reliability and Validity. J. Appl. Soc. Psychol..

[30] Cox K.N., Giglia R.C., Binns C.W. (2015). The Influence of Infant Feeding Attitudes on Breastfeeding Duration: Evidence from a Cohort Study in Rural Western Australia. Int. Breastfeed. J..

[31] Twells L.K., Midodzi W.K., Ludlow V., Murphy-Goodridge J., Burrage L., Gill N., Halfyard B., Schiff R., Newhook L.A. (2016). Assessing Infant Feeding Attitudes of Expectant Women in a Provincial Population in Canada: Validation of the Iowa Infant Feeding Attitude Scale. J. Hum. Lact..

[32] Abdulahi M., Fretheim A., Argaw A., Magnus J.H. (2020). Adaptation and Validation of the Iowa Infant Feeding Attitude Scale and the Breastfeeding Knowledge Questionnaire for Use in an Ethiopian Setting. Int. Breastfeed. J..

[33] Cox J.L., Holden J.M., Sagovsky R. (1987). Detection of Postnatal Depression: Development of the 10-Item Edinburgh Postnatal Depression Scale. Br. J. Psychiatry.

[34] Mahmud W.M.R.W., Awang A., Mohamed M.N. (2003). Revalidation of the Malay Version of the Edinburgh Postnatal Depression Scale (EPDS) among Malay Postnatal Women Attending the Bakar Bata Health Center in Alor Setar, Kedah, North West of Peninsular Malaysia. Malays. J. Med. Sci..

[35] Arifin S.R.M., Ahmad A., Rahman R.A., Loh H.S., Ng C.G. (2014). Postpartum Depression in Malaysian Women: The Ssociation with the Timing of Pregnancy and Sense of Personal Control during Childbirth. Int. J. Acad. Res..

[36] Shukri N.A.M. (2018). Exclusive Breastfeeding: Knowledge, Attitude, Practice, and Its Determinants among Malay Mothers in Ampang, Selangor. Int. J. Allied Health Sci..

[37] Yu J., Wei Z., Lukoyanova O., Borovik T., Fewtrell M.S. (2020). Maternal Infant-Feeding Attitudes, Infant Eating Behaviors, and Maternal Feeding Choice at 3 and 6 Months Postpartum: A Comparative Multicenter International Study. Breastfeed. Med..

[38] Ishak S., Adzan N.A.M., Quan L.K., Shafie M.H., Rani N.A., Ramli K.G. (2014). Knowledge and Beliefs About Breastfeeding Are Not Determinants for Successful Breastfeeding. Breastfeed. Med..

[39] Arifunhera J.H., Srinivasaraghavan R., Sarkar S., Kattimani S., Adhisivam B., Vishnu Bhat B. (2016). Is Maternal Anxiety a Barrier to Exclusive Breastfeeding?. J. Matern.-Fetal Neonatal Med..

[40] Abiama E.E., Ezeh S.O. (2020). Influence of Postpartum Depression and Maternal Employment Status on Attitude towards Exclusive Breast Feeding. Niger. J. Psychol. Res..

[41] Briere C.E., McGrath J.M., Cong X., Brownell E., Cusson R. (2016). Direct-Breastfeeding in the Neonatal Intensive Care Unit and Breastfeeding Duration for Premature Infants. Appl. Nurs. Res..

[42] Lara-Cinisomo S., McKenney K., Di Florio A., Meltzer-Brody S. (2017). Associations between Postpartum Depression, Breastfeeding, and Oxytocin Levels in Latina Mothers. Breastfeed. Med..

[43] Pope C.J., Mazmanian D. (2016). Breastfeeding and Postpartum Depression: An Overview and Methodological Recommendations for Future Research. Depress. Res. Treat..

[44] Figueiredo B., Dias C.C., Brandão S., Canário C., Nunes-Costa R. (2013). Breastfeeding and Postpartum Depression: State of the Art Review. J. Pediatr..

[45] Islam M.J., Broidy L., Baird K., Rahman M., Zobair K.M. (2021). Early Exclusive Breastfeeding Cessation and Postpartum Depression: Assessing the Mediating and Moderating Role of Maternal Stress and Social Support. PLoS ONE.

[46] Khalifa D.S., Glavin K., Bjertness E., Lien L. (2016). Determinants of Postnatal Depression in Sudanese Women at 3 Months Postpartum: A Cross-Sectional Study. BMJ Open.

[47] Liang P., Wang Y., Shi S., Liu Y., Xiong R. (2020). Prevalence and Factors Associated with Postpartum Depression during the COVID-19 Pandemic among Women in Guangzhou, China: A Cross-Sectional Study. BMC Psychiatry.

[48] Suárez-Rico B.V., Estrada-Gutierrez G., Sánchez-Martínez M., Perichart-Perera O., Rodríguez-Hernández C., González-Leyva C., Osorio-Valencia E., Cardona-Pérez A., Helguera-Repetto A.C., Sosa S.E.Y. (2021). Prevalence of Depression, Anxiety, and Perceived Stress in Postpartum Mexican Women during the Covid-19 Lockdown. Int. J. Environ. Res. Public Health.

[49] Davenport M.H., Meyer S., Meah V.L., Strynadka M.C., Khurana R. (2020). Moms Are Not OK: COVID-19 and Maternal Mental Health. Front. Glob. Women’s Health.

[50] Myers S., Emmot E.H. (2021). Postnatal depression symptom trajectories across the COVID-19 pandemic: Evidence from the United Kingdom. OSF Prepr..

[51] Suhana Yahya N.F., Teng N.I.M.F., Das S., Juliana N. (2020). Postpartum Depression among Neonatal Intensive Care Unit Mothers and Its Relation to Postpartum Dietary Intake: A Review. J. Neonatal Nurs..

[52] Pisoni C., Spairani S., Manzoni F., Ariaudo G., Naboni C., Moncecchi M., Balottin U., Tinelli C., Gardella B., Tzialla C. (2019). Depressive Symptoms and Maternal Psychological Distress during Early Infancy: A Pilot Study in Preterm as Compared with Term Mother-Infant Dyads. J. Affect. Disord..

[53] Pacheco F., Sobral M., Guiomar R., de la Torre-Luque A., Caparros-Gonzalez R.A., Ganho-ávila A. (2021). Breastfeeding during Covid-19: A Narrative Review of the Psychological Impact on Mothers. Behav. Sci..

[54] Hamid S.B.A., Chih H.J., Binns C. (2017). Predictors of Breastfeeding Intention in Malaysia. Environ.-Behav. Proc. J..

[55] Abekah-Nkrumah G., Antwi M.Y., Nkrumah J., Gbagbo F.Y. (2020). Examining Working Mothers’ Experience of Exclusive Breastfeeding in Ghana. Int. Breastfeed. J..

[56] Soomro J.A., Shaikh Z.N., Saheer T.B., Bijarani S.A. (2016). Employers’ Perspective of Workplace Breastfeeding Support in Karachi, Pakistan: A Cross-Sectional Study. Int. Breastfeed. J..

[57] Seah S.S.Y., Cheah F.C. (2017). Empowering Mothers of Preterm Infants for Continuous Breastfeeding in Malaysia. Perinatology.

